# Variations in the Home Language Environment and Early Language Development in Rural China

**DOI:** 10.3390/ijerph18052671

**Published:** 2021-03-06

**Authors:** Yue Ma, Laura Jonsson, Tianli Feng, Tyler Weisberg, Teresa Shao, Zixin Yao, Dongming Zhang, Sarah-Eve Dill, Yian Guo, Yue Zhang, Dimitris Friesen, Scott Rozelle

**Affiliations:** 1 Rural Education Action Program, Freeman Spogli Institute for International Studies, Stanford University, Palo Alto, CA 94305-6055, USA; yma3@stanford.edu (Y.M.); Laura.c.jonsson@gmail.com (L.J.); tweisberg22@groton.org (T.W.); teresa01px2022@saschina.org (T.S.); syao@thacher.org (Z.Y.); dongming@stanford.edu (D.Z.); sedill@stanford.edu (S.-E.D.); yg1047@stanford.edu (Y.G.); dfriesen@stanford.edu (D.F.); rozelle@stanford.edu (S.R.); 2 School of Management and Economics, University of Electronic Science and Technology of China, Chengdu 610054, China; 3 Child Health Care Department, National Center for Women and Children’s Health, Chinese Center for Disease Control and Prevention, Beijing 100081, China; zhangyue0416@163.com

**Keywords:** home language environment, child development, language development, rural China, individual differences

## Abstract

The home language environment is critical to early language development and subsequent skills. However, few studies have quantitatively measured the home language environment in low-income, developing settings. This study explores variations in the home language environment and child language skills among households in poor rural villages in northwestern China. Audio recordings were collected for 38 children aged 20–28 months and analyzed using Language Environment Analysis (LENA) software; language skills were measured using the MacArthur–Bates Mandarin Communicative Developmental Inventories expressive vocabulary scale. The results revealed large variability in both child language skills and home language environment measures (adult words, conversational turns, and child vocalizations) with 5- to 6-fold differences between the highest and lowest scores. Despite variation, however, the average number of adult words and conversational turns were lower than found among urban Chinese children. Correlation analyses did not identify significant correlations between demographic characteristics and the home language environment. However, the results do indicate significant correlations between the home language environment and child language skills, with conversational turns showing the strongest correlation. The results point to a need for further research on language engagement and ways to increase parent–child interactions to improve early language development among young children in rural China.

## 1. Introduction

The home language environment is critical to early childhood development and long-term outcomes. The literature has shown that a child’s home language environment is a strong predictor of early language skills [1,2,3]. Specifically, children growing up in households with richer home language environments have been shown to learn vocabulary faster, exhibit increased processing speed, and develop overall stronger language and cognitive skills [4,5,6]. Better early language skills, in turn, have been identified as determinants of future academic outcomes [7,8].

Past research has found significant variation in the home language environment and in the development of early language skills among socioeconomic groups. Studies consistently have found that low socioeconomic status (SES) families tend to have less robust home language environments than high SES families [2,3,6,9,10,11,12]. Studies have found that lower SES households tend to talk less and use less-varied vocabulary with their children than higher SES households [4,9,13,14], as well as engage in less child-directed speech (CDS) [2,15]. In part due to a lack of diversity in language, researchers have found that children from low-SES backgrounds tend to have less-developed language skills and overall cognitive abilities than their higher SES counterparts [16]. Fernald et al. [8], for example, found that the English language processing efficiency of 24-month-old infants from lower SES families was similar to 18-month-old children from higher-SES families, meaning there was a 6-month gap between SES groups in processing skills critical to language development.

In addition to variations among socioeconomic groups, there is preliminary evidence of variations in the home language environment and early language skill development among families within the same SES group. In a study of 29 Spanish-learning infants from low-SES families, Weisleder and Fernald [2] found striking variability in the amount of overheard speech and CDS recorded. This study also found that the diverse amount of CDS heard correlated to word processing abilities within the sample that was shown to have relatively homogenous demographic characteristics. Weisleder and Fernald [2] also posit that despite the proven link between factors related to SES and the home language environment, variability in parental verbal engagement is also due in part to factors independent of social class.

Despite the literature on the variation in home language environments and language skills among and within socioeconomic groups, the majority of research on the home language environment measured with audio recording technology focuses on samples from high-SES and Western settings. Two recent systematic reviews of Language Environment Analysis (LENA) audio recording technology [11,17] found that there are only two studies focused on a non-Western sample, including one study from urban Shanghai [18] and one from South Korea [19]. In our own literature review, we found two additional studies from non-Western settings: Weber et al. [20] in Senegal, and Casillas et al. [21] in Mexico. The study from Mexico found that despite limited CDS, children displayed no delay as compared to their Western counterparts [21]; however, in Senegal, Weber et al. [20] found that marked improvements could be made to CDS and child utterances. Moreover, although the other two studies were carried out in East Asian countries (China and South Korea), both of the studies targeted high-SES populations [18,19].

There is also some evidence from low-SES populations in low- and middle-income countries (LMICs) on the home environment more generally. Past studies have utilized evidence from non-Western, developing settings to understand family care for children and parenting practices, finding overall low levels of interactive parenting (such as singing, reading, and playing) at home [22]. However, these studies have typically relied on self-report measurements, such as the Family Care Indicators [23,24,25]. While such measures are useful, they may be subject to self-report bias. Objective, quantitative measures of the home language environment are therefore needed to examine variations in language investments and identify factors linked to child language development in LMICs, where approximately 250 million children are at risk of developmental delays [26].

Considering this gap in the literature, rural China provides a unique opportunity to examine empirically the home language environment and factors driving its variation in a non-Western, developing setting. The literature has found that rural China, like other LMICs, faces high rates of early developmental delays [23,24,25]. In a study of 3353 children under three years across rural China, 85% were found to have at least one developmental delay, and 52% were found to have delayed language skills specifically [25]. The literature also has found that the observed high rates of developmental delays, especially language delays, are strongly linked to low levels of interactive parenting (playing, singing, and telling stories/reading) by caregivers at home [23,24,25]. These low rates of interactive parenting practices found in rural China are similar to the rates found in a study of 28 other LMICs [27]. However, while studies have identified poor parenting practices as one of the sources of poor developmental outcomes, no study has quantitatively measured the home language environment among rural households in China, which could lend insights into improving the early language skills of children in rural China and other LMICs.

Overall, this study aims to provide a descriptive picture of the home language environment and language skill development outcomes of children in rural Shaanxi, China. To do so, we have four specific objectives. The first objective of this study is to quantitatively characterize the distribution of child language skills. The second objective is to illustrate the quality and heterogeneity of home language environments. The third objective is to reveal the characteristics related to high and low levels of investment in the home language environment. The fourth and final objective is to compare the language skills of children in households that invest in the home language environment to different extents.

## 2. Materials and Methods

### 2.1. Sample Selection

**Figure 1:**
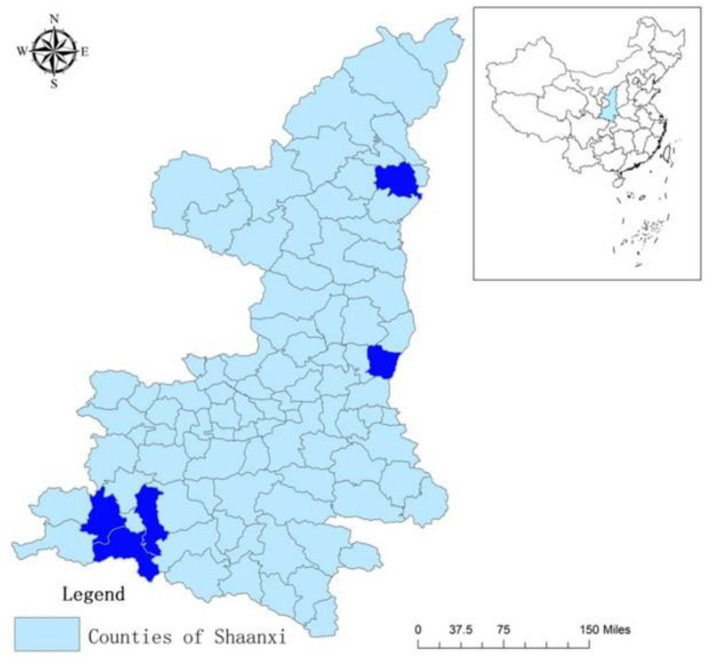
Study counties (dark blue) in Shaanxi province, China. Study counties clockwise from top: Suide, Heyang, Nanzheng, Chenggu, Mianxian. Source: Zhou et al. [29]. Sample counties colored using Adobe Photoshop (Adobe Inc., San Jose, CA, USA).

Data for this study were collected in 2019 in five counties of Shaanxi Province. Geographically situated in northwest China, Shaanxi ranks 19 out of the 31 provinces in terms of Gross Domestic Product (GDP) per capita, with a per capita income of approximately $8000 in 2015 [28]. Geographically, we selected counties that represent southern, central and northern Shaanxi. Three of the five sample counties are nationally designated as poverty-stricken counties. The populations in all five sample counties are 99% Mandarin-speaking Han Chinese [28]. Most households speak their own local dialect at home, however most dialects are very similar to Mandarin. Figure 1 displays sample county locations within Shaanxi Province.

After choosing the sample counties, we followed a three-step protocol to select participants in the sample. First, one township was randomly selected from within each county. Next, one village from each township was randomly selected. If the village had fewer than eight toddlers in our desired age range (20–27 months), the team selected neighboring villages from the same township until a total of eight toddlers per township was reached. Finally, all families with children in our desired age range were included in the study sample. In total, the sample includes 38 rural, Mandarin-speaking families with children from 16 villages. After sampling was carried out, families were approached in their homes by members of the enumeration team. The enumeration team followed a set script and collected oral consent from each family, instructing them that their participation was voluntary, and that they could withdraw consent or discontinue participation at any time.

### 2.2. Measures

#### 2.2.1. Home Language Environment: LENA

To quantitatively measure the home language environment in our sample, we utilized the Language Environment Analysis (LENA) recording system [30,31]. Each LENA recorder is capable of capturing a 16 h audio recording of a typical day at home for a participant, enabling unobtrusive recording of the child’s natural home language environment. LENA has previously been shown to be valid and reliable in American English, Spanish, French, Korean, Dutch, Vietnamese, and Swedish when compared with trained human transcribers [19,32,33,34,35,36]. LENA technology has also been adapted and validated for Mandarin-speaking populations [18,33].

In our study, we asked each participating household to make one 16 h recording of their home language environment. The recording was scheduled to be completed on a “normal day” for the household, meaning a day that is representative of the child’s typical at-home experience. In rural villages in China, childcare services are non-existent, and thus caregivers are usually mothers and grandmothers who stay at the household throughout the day. Caregivers stay with the child at home most of the time during a “typical day,” but may also visit neighbors. Following the LENA validation protocols, the recorder was placed in the chest pocket of a specialized shirt that the child wore throughout the day. Families were instructed to start the recording in the morning and only remove the recorder when the child bathed or slept for the night. The next day, our team returned to collect the recorders and follow up with participants.

Completed home recordings were processed by the LENA analysis software, which processed the audio files and yielded three automated measures of the home language environment: Adult Word Count (AWC), Conversational Turn Count (CTC), and Child Vocalization Count (CVC). The AWC measures the number of adult words spoken in the vicinity of the child. It should be noted, however, that in this context, measured AWC does not distinguish between child-directed speech and speech in the vicinity of the child; the CTC is a measure of adult-child alternations in conversation; and the CVC measures the number of pre-speech (non-word communicative sounds such as squeals, growls, or raspberries) or speech productions (words or babbles) made by the participating child [32]. The importance of AWC, CTC and CVC in determining the nature of child language skills and the home language environment has been noted in multiple studies [37,38,39].

After collecting the audio data, we standardized our 16 h recordings into 12 h data due to variations in start time between families. To adjust for skewing commonly seen with count data, we applied five steps of adjustments. First, we used Chebyshev polynomials transformation to normalize the distribution. Second, least absolute shrinkage and selection operator (LASSO) regression models were used to select the final Chebyshev polynomials model used in transforming the data. Third, residuals were predicted with the final Chebyshev polynomials model. Fourth, residualized count variables were estimated from these transformed data and then rescaled back to the original count metric. Outcomes (AWC, CTC and CVC) were totals from the first usable 12 h recordings of the participants.

#### 2.2.2. Child Language Skills and Demographic Information

Beyond the LENA measurement, we also administered the Mandarin version of the MacArthur-Bates Communicative Development Inventories (CDI) to the family. The CDI is a parent-report assessment adapted and validated in Mandarin Chinese (MCDI) [40,41]. The MCDI that we utilized was the expressive vocabulary assessment for children between 16 and 30 months. This scale measured the number of words that a child could or could not say from an inventory of 113 words in total. For words a child could say, enumerators periodically asked the primary caregiver of the child to provide the context in which the child used the word. The status of the child’s developmental progress was obtained by comparing the results with empirically-determined cutoff scores established by Tardif et al. [41]. Rates of delay were first established for each age group according to that group’s specific cutoff score (any child under the 10th percentile of language development in their one-month age group was considered delayed); these were then combined to give a rate of delay for the entire sample.

We also collected demographic information from the caregivers of all households using an enumerator-administered survey form. This survey included questions that allowed us to document child and family characteristics for each household At the child level, we included child age, gender and premature birth status [5]. We also collected data related to household socioeconomic status, as it has been shown to be associated with language development [2,3,6,42], including parental age, education level, whether the parents were present in the household for the majority of the previous year, whether the parents currently lived at home with the sample child and the value of household assets. To measure the value of household assets, we created a family asset index for all households using polychoric principal components analysis based on whether the family owned or had access to running water, a toilet, a water heater, a washing machine, a computer, Internet access, a refrigerator, air conditioning, a motorcycle, and a car/truck [43]. Additionally, we collected data on the number of siblings and adults in the household, as it has been suggested that household size is an influential factor in the home language environment and language development [44,45].

### 2.3. Statistical Analysis

Our statistical analysis consists of three parts. To describe the heterogeneity of the home language environment, we first graphically present the AWC, CTC, and CVC scores for the household in the sample in rank order. We then graphically present the variation in Mandarin CDI expressive vocabulary scores to describe the nature of child language skills. We present the means, standard deviations, and ranges for both the LENA and CDI distributions.

To analyze correlations between participant demographic characteristics and the home language environment we performed a *t*-test comparing the characteristics of households that ranked in the top and bottom terciles of each of the three measures of the home language environment (AWC, CTC and CVC). We conduct these *t*-tests to explicitly examine the bottom tercile of children within our sample, as we believe that these children should be the focus of future policy regarding the home language environment in rural China. To examine the entire sample, we additionally include multivariate analysis between child and family characteristics and the three measures of the home language environment (AWC, CTC and CVC).

Finally, to analyze the correlations between the home language environment and child language skills, we conduct an additional *t*-test comparing the CDI scores of children in the top and bottom terciles of AWC, CTC, and CVC, for the same reasons described above. Again, to examine the entire sample, we include multivariate analysis between the three measures of the home language environment and CDI scores. All statistical analyses were performed using STATA 16.1 (StataCorp, College Station, TX, USA).

## 3. Results

The descriptive statistics from the survey of the sample households are shown in Table 1. Of the 38 sample children, the average age was 25 months old. Just over half (53%) were male, and 11% were born prematurely. Regarding household characteristics, the average age of mothers was 29 years old. About 21% of mothers reported completing high school or above, whereas only 11% of fathers had completed high school. In 74% of the households, the mother was the primary caregiver; in the other 26% of households, the paternal grandmother was most often the primary caregiver. In 63% of households, the father lived at home for most of the previous year. The average number of adults in each household was 2, and 26% of children (10/38) had siblings.

**Figure 2:**
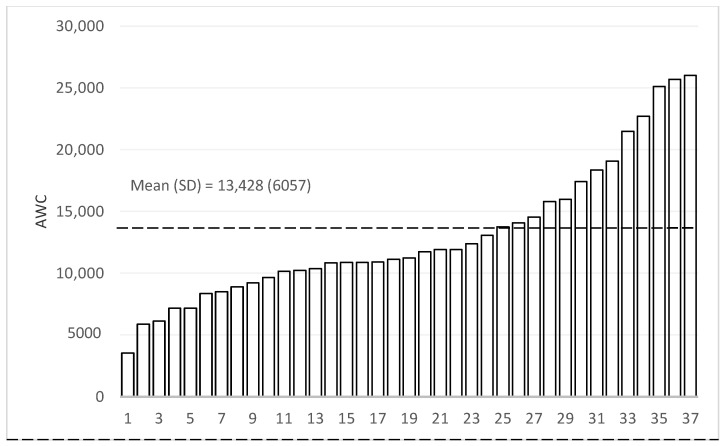
Distribution of Adult Word Count (AWC, N = 38). “SD” is defined as standard deviation.

**Figure 3:**
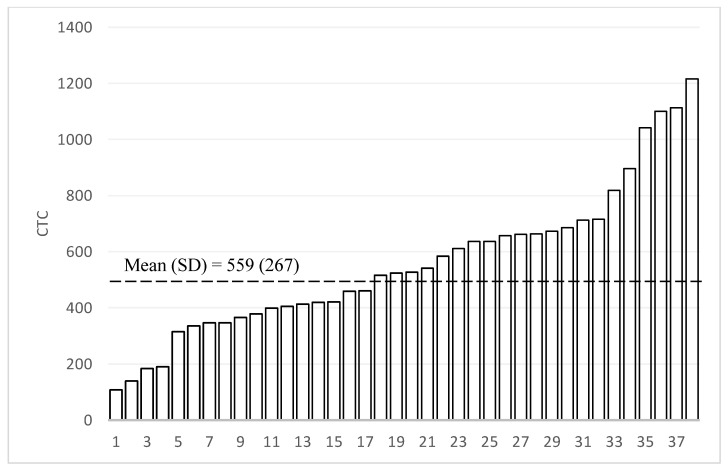
Distribution of Conversational Turn Count (CTC, N = 38). “SD” is defined as standard deviation.

**Figure 4:**
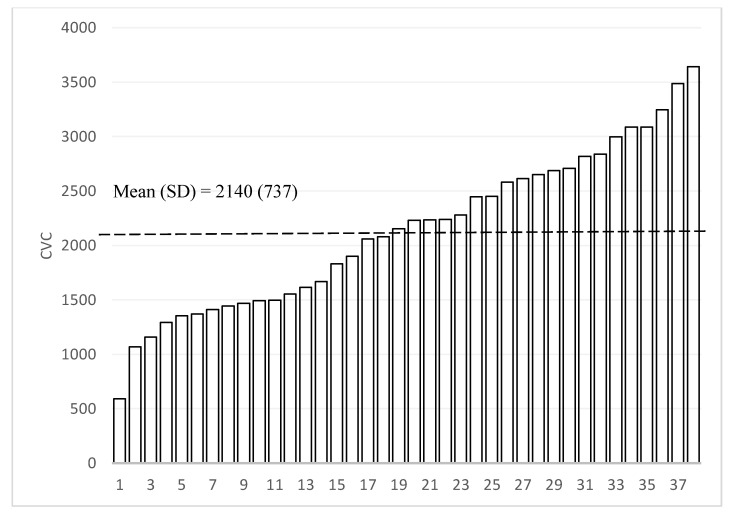
Distribution of Child Vocalization Count (CVC, N = 38). “SD” is defined as standard deviation.

Figure 2, Figure 3 and Figure 4 present the measures of the home language environment among sample children (Adult Word Count (AWC), Conversational Turn Count (CTC), and Child Vocalization Count (CVC), respectively). The average AWC of the sample was 13,428 words (SD = 6057—Figure 2). When looking at the heterogeneity of the sample, there was a 5-fold difference between the highest (AWC = 27,949) and lowest scoring participant (AWC = 3542). The mean CTC was 559 (SD = 267), and the difference between the highest and lowest participants was over six-fold (Figure 3). The average CVC was 2140 (SD = 737), and the difference in CVC between the highest and lowest values was around five-fold (Figure 4).

**Figure 5:**
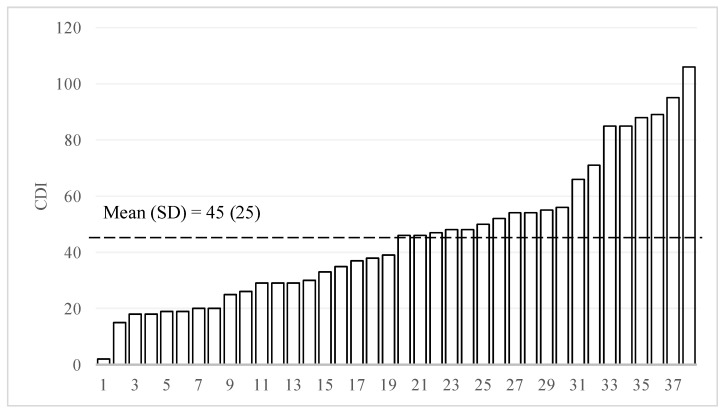
Distribution of MacArthur–Bates Communicative Development Inventories scores (CDI, N = 38). “SD” is defined as standard deviation.

Figure 5 presents the MacArthur–Bates Communicative Development Inventories (CDI) scores of children in the sample. The mean CDI score was 45, with a standard deviation of 25. When looking at the heterogeneity of the sample, there was a 53-fold difference between the highest (CDI = 106) and lowest score (CDI = 2) among sample children. Hence, similar to the results in Figure 2 through Figure 4, the variation in CDI scores between the 38 individual different samples was large.

Table 2, Table 3 and Table 4 present the results of our descriptive *t*-tests comparing demographic characteristics of households that make up the top and bottom terciles of our sample in terms of AWC, CTC, and CVC, respectively. Across the three tables, there were no statistically significant differences at the 5% level or above for any of the language skill measures. Furthermore, most demographic characteristics do not show a consistent trend in their relationship with the home language environment measures. For example, maternal education was somewhat higher in the bottom terciles of AWC and CTC, but lower in the bottom tercile of CVC. Two exceptions (although not statistically significant) were that (1) families ranking in the lower terciles of AWC, CTC, and CVC consistently showed a higher likelihood of older maternal age and higher paternal education, and (2) families ranking in the lower terciles of AWC, CTC, and CVC consistently showed a higher likelihood of having more children in their household.

The multivariate analysis of the relationship between child and family characteristics and the home language environment (AWC, CTC and CVC) is shown in Table 5. Consistent with the results from our univariate analyses, we find no significant correlations between child or family characteristics and AWC/CVC in multivariate models. When controlling for possible confounders, older mothers (*p*-value = 0.046) are significantly associated with higher CTC scores.

**Figure 6:**
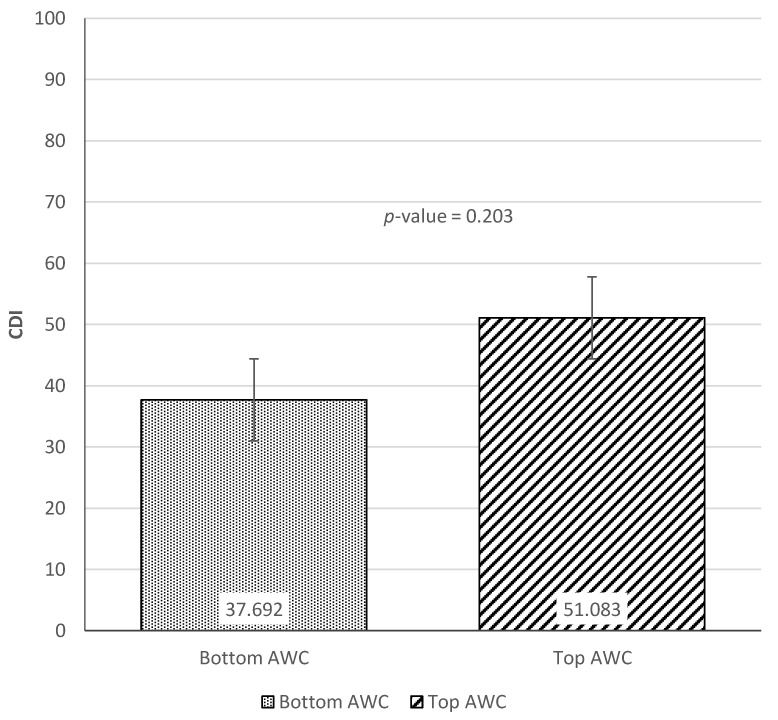
Difference in Communicative Development Inventories scores between top and bottom terciles of Adult Word Count.

**Figure 7:**
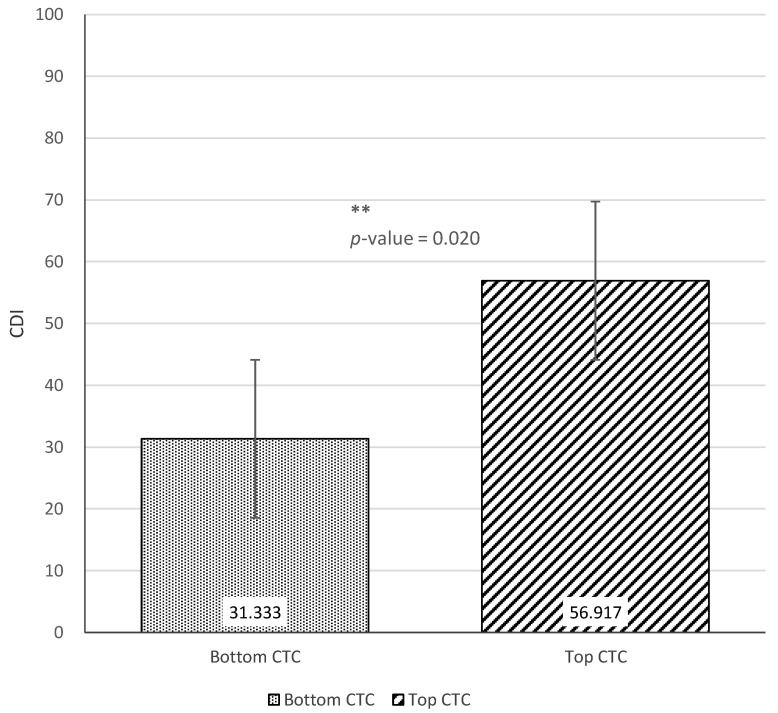
Difference in Communicative Development Inventories scores between top and bottom terciles of Conversational Turn Count. ** *p* < 0.05.

**Figure 8:**
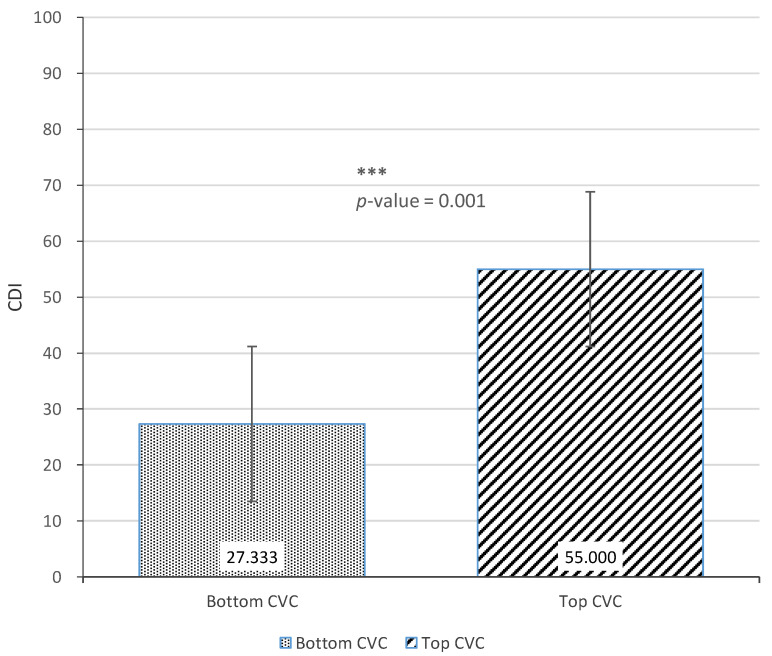
Difference in Communicative Development Inventories scores between top and bottom terciles of Child Vocalization Count. *** *p* < 0.01.

Figure 6, Figure 7 and Figure 8 show the results of the *t*-tests comparing child language skills between the top and bottom terciles of each home language environment measure (AWC, CTC, and CVC). Figure 6 presents the difference in CDI scores between the top and bottom terciles of AWC. Although not statistically significant (*p*-value = 0.203), there was a difference of 13.4 CDI points between the top and bottom tercile of AWC. Figure 7 next presents the difference between the CDI scores of children in the top and bottom terciles of CTC. The results find a difference of 15.6 CDI points between the two groups, with children in the top tercile of CTC showing significantly stronger language skills (*p*-value = 0.020). Similarly, Figure 8 finds that children in the top tercile of CVC scored 17.7 CDI points higher than children in the bottom tercile, significant at the 1% level (*p*-value = 0.001). Taken together, the results indicate positive correlations between the measures of home language environment and child language skills.

Table 6 shows the multivariate correlation analysis between our measure of child language development (CDI) and our three indicators of the home language environment (AWC, CTC and CVC). We find a positive and significant correlation between CDI and CTC, (*p*-value = 0.033), but no significant correlation between CDI and AWC (*p*-value = 0.589) and CVC (*p*-value = 0.083). Despite the fact that this correlation is positive and significant, it is also relatively small.

## 4. Discussion

This study presents the first-ever findings on variation in the home language environment and language skills of young children from poor rural households in China, using data from children age 20–27 months in rural Shaanxi, China. The results found striking variation in all home language environment measures, including Adult Word Count (AWC), Conversational Turn Count (CTC) and Child Vocalization Count (CVC). The only demographic characteristic that was associated with the home language environment was maternal age, which was positively associated with CTC score. Our analysis also revealed a significant association between one measure of the home language environment (CTC) and child language skills (MacArthur–Bates Communicative Development Inventories (CDI) scores).

Evidence from this study indicates that the children in our low-SES rural China sample have substantially less diversity in their home language environments than children in higher SES families. On average, 20–27 month old children in our sample heard 14,739 adult words (AWC), engaged in 611 conversational turns (CTC), and made 2332 vocalizations (CVC) throughout the 16-h recordings. In comparison, a sample of even younger children in urban Shanghai heard 28,131 words and engaged in 1001 conversational turns [18]. Moreover, since children in the urban sample are roughly one year younger than this rural sample, these differences should be considered as lower bound estimates of the home language environment gap between urban and rural children in China.

When we compare the results from our study to other non-Western settings, we find several interesting differences. Compared to the study from Pae et al. [19] in South Korea, we find that the children from our study were only slightly behind. The children from South Korea had a measured AWC of 14,053 and a CTC of 377. Since these children were 10 months old, and CTC increases by about 17.6 conversational turns per month between 10–24 months [46], we find an extrapolated 24-month old CTC of 624. The South Korean AWC (14,053) and CTC (624) are only slightly higher than our measured AWC (13,428) and CTC (559). This difference may be partially due to the fact that the South Korean sample included children from higher-SES families. However, a study by Weber et al. [20] in Senegal included low-SES families that may be more comparable to our study sample. These families were also ahead of those in our sample in terms of home language environment measures. The measured CTC and CVC in the sample from Senegal, after extrapolating hourly data into a 12 h comparable total, were 654 and 2640, respectively. These are again slightly higher than that of our sample (559 CTC, 2140 CVC).

Despite the homogeneity of our study sample (in terms of their household characteristics), the results found striking variation within the sample for all measures of the home language environment. There was a 5-fold difference between the highest and lowest ranking child in terms of the AWC and CVC measures, and a 6.6-fold difference in CTC scores. This heterogeneity is consistent with findings in a paper by Weisleder and Fernald [2], where the authors found an even greater difference (15-fold) in AWC scores among low-SES Spanish-learning infants in the United States. The result also appears to indicate that variation in the home language environment among low-SES households (that is, within the sample) is present in diverse countries and linguistic contexts. This is supported by findings from Suskind et al. [47] that the home language environment in low-SES households is quite malleable, as shown by a parent-directed language intervention. These early gaps in the home language environment are concerning, as research indicates that home language environments in early childhood not only impact a child’s language development in the short run but also lead to differences in cognitive ability and reading literacy in later childhood [8,48].

Similar to the findings on the home language environment, the study found a high prevalence of delays in language skills (CDI) and high levels of variation in the CDC scores of children in low-SES households in rural northwestern China. About two-thirds (58%) of our sample were below the Mandarin CDI cutoff for typical language development. However, there was large variation in CDI scores across the sample, providing further evidence for the heterogeneity we see in early language learning for children in lower-SES communities. Given that such a large share of rural children is at risk for delayed language development, this finding poses a grave concern for China’s rural development, as poor development of early language skills can limit the development of future skills and achievement [1].

Our sample does not provide strong evidence that investment in the home language environment is closely related to specific household characteristics or conditions. In this way, the findings in this paper deviate from the literature that shows that families with better-educated mothers typically provide richer home language environments to their children [5,9]. Instead, our results suggest that no identifiable demographic characteristic can explain the variability of the home language environment of rural children. The lack of significant associations may be explained by the fact that we are studying a relatively homogeneous population of low-SES families in rural China. However, larger sample sizes in future studies may illuminate potential associations.

Finally, the results demonstrate that the home language environment is positively and significantly correlated with child language skills. Sample children in the top terciles of AWC, CTC, and CVC all showed higher CDI scores compared to children in the bottom terciles; this difference was statistically significant for both the CTC and CVC scores. The lack of significance for AWC may be because the AWC measure included all speech that was overheard within 6 to 10 feet of the child, including speech that was not directed toward the child. If the AWC measure were to include child-directed speech only, it is possible, and indeed likely, that this result would become significant. This is supported by the finding that CTC, which measures verbal interaction between the child and caregiver, was most strongly associated with CDI scores, underscoring the importance of child-directed speech and caregiver–child interaction in early language development.

These findings point to an urgent need for policymakers to invest in broadly delivered programs to improve the home language environment and stimulate child language development in low-income rural communities in China. Our finding that conversational turns were most strongly correlated with language skills indicates that these programs should focus on promoting the importance of caregiver–child interaction and teaching caregivers how to engage in stimulating, age-appropriate conversations with their children. Investment in effective parental training to increase parental engagement and interaction with their children may improve child language skills, as well as cognitive and non-cognitive development. The Language Environment Analysis (LENA) recording device may also be a potential training tool, as indicated by studies that have used LENA recordings in interventions to improve parental stimulation and child language development [18,47,49].

This is one of the first studies to quantitatively examine the home language environment in a low-SES population in a developing setting, and it is the first study to do some among a low-SES community in China. The findings of this study add to the body of knowledge of the home language environment and child language skills in LMICs, where an estimated 250 million children are at risk for not achieving their developmental potential [26]. This study also sheds light on the high rates of developmental delays among China’s rural infants and toddlers: our results indicate that despite a high degree of variation, measures of the home language environment among households in rural China are significantly lower than in urban China, which corresponds to the observed gaps between rural and urban children in early language and cognitive skills [25].

We also acknowledge two limitations of this study. First, typical to nearly all existing studies of the home language environment, particularly those using LENA technology, our sample size is relatively small. This is due to the logistical complexity of implementing LENA recordings in a rural area like Shaanxi province where villages and townships are widely distributed. As this is the first use of LENA recordings in rural China, we view this as a pilot study. Despite this limitation, the sample was large enough to identify significant variations in the home language environment among the sample and capture correlations with child language outcomes. Future research with larger sample sizes, including our own ongoing studies, may provide further evidence of demographic characteristics associated with caregiver investments and child language skills in rural China. Second, our AWC data did not distinguish the quantity of overheard speech versus child-directed speech, which is more highly predictive of language outcomes and long-term learning [2]. For this reason, we must caution against making generalizations based on overheard speech vs. child-directed speech in the context of this study. We would expect to see stronger and statistically significant correlations between child-directed speech and CDI in our sample, and future studies should attempt to distinguish between overheard speech and child-directed speech to more accurately identify drivers of infant language skill development. Ultimately, this study points to a need for further research to determine how to improve the home language environment and child language skills development in rural China and LMICs more broadly.

## 5. Conclusions

This study presents the first-ever exploration of the home language environment and language skills in poor areas of rural China. We analyzed audio recordings of 38 children aged 20–28 months employing Language Environment Analysis (LENA) software and measured language skills using the MacArthur–Bates Mandarin Communicative Developmental Inventories (CDI) expressive vocabulary scale. We found that both CDI scores and measures of the home language environment including Adult Word Count (AWC), Conversational Turn Count (CTC) and Child Vocalization Count (CVC) were widely distributed throughout our sample. We did not find significant correlation between demographic characteristics and the home language environment, but we did find significant and positive correlations between the home language environment and child language skills, the strongest of which being conversational turns. We also found considerable variation in the home language environment and child language skills within our sample with differences between high and low scores of 5 to 6 times. Further research is needed on language engagement and methods to increase parent–child interactions, as this can improve language development among young children in rural China.

## Figures and Tables

**Table 1:** Descriptive statistics of child and household characteristics (N = 38).

Variables	Frequency/Mean (N = 38)
***Child characteristics***	
Age, months, Mean (SD)	24.58 (2.07)
Male, *n* (%)	20 (52.63)
Premature, *n* (%)	4 (10.53)
***Household characteristics***	
Age of mother, years, Mean (SD)	28.76 (3.66)
Mother completed middle school or above, *n* (%)	8 (21.05)
Mother is primary caregiver, *n* (%)	28 (73.68)
Number of adults in household, Mean (SD)	2.08 (1.08)
Number of siblings in household, Mean (SD)	0.26 (0.45)
Father completed middle school or above, *n* (%)	4 (10.53)
Father lived at home during most of last year, *n* (%)	24 (63.16)
Asset index (PCA score), Mean (SD)	0.00 (1.77)

Source: Authors’ survey. Note: In our survey, the education level of both the mother and father is recorded as a binary variable equal to one if that parent completed middle school or above, or 0 if they did not. Completion of middle school requires 9 years of total schooling and is the last stage of compulsory/free education in China. “n” is defined as frequency. “SD” is defined as Standard Deviation. “PCA” is defined as Principal Component Analysis.

**Table 2:** Differences in characteristics between top and bottom terciles of Adult Word Count (AWC, N = 38).

Variables	Bottom ⅓ of AWC	Top ⅓ of AWC	Difference
	(1)	(2)	(3) = (2) − (1)
***Child characteristics***			
Age (months)	24.885	23.775	−1.110
	(2.225)	(2.024)	(0.853)
Gender (1 = boy)	0.385	0.500	0.115
	(0.506)	(0.522)	(0.206)
Prematurity (1 = yes)	0.154	0.000	−0.154
	(0.376)	(0.000)	(0.109)
***Household characteristics***			
Age of Mother (years)	27.615	30.000	2.385
	(3.595)	(4.134)	(1.546)
Maternal education (1 = completed high school or above)	0.308	0.167	−0.141
	(0.480)	(0.389)	(0.176)
Mother is primary caregiver (1 = yes)	0.692	0.667	−0.026
	(0.480)	(0.492)	(0.195)
Paternal education (1 = completed high school or above)	0.077	0.167	0.090
	(0.277)	(0.389)	(0.134)
Father lived at home during most of last year (1 = yes)	0.769	0.667	−0.103
	(0.439)	(0.492)	(0.186)
Asset index (PCA score)	0.109	−0.533	−0.642
	(2.168)	(1.399)	(0.737)
Number of adults in the household	1.923	2.167	0.244
	(1.188)	(1.193)	(0.477)
Number of siblings at home	0.308	0.250	−0.058
	(0.480)	(0.452)	(0.187)

Standard errors in parentheses; None of the results in this table are significant. “AWC” is defined as Adult Word Count. “PCA” is defined as Principal Component Analysis. Column (3) represents the value of Column (2) minus Column (1).

**Table 3:** Differences in characteristics between top and bottom terciles of Conversational Turns Count (CTC, N = 38).

Variables	Bottom ⅓ of CTC	Top ⅓ of CTC	Difference
	(1)	(2)	(3) = (2) − (1)
***Child characteristics***			
Age (months)	24.686	24.336	−0.350
	(2.325)	(1.907)	(0.868)
Gender (1 = boy)	0.333	0.667	0.333
	(0.492)	(0.492)	(0.201)
Prematurity (1 = yes)	0.083	0.083	−0.000
	(0.289)	(0.289)	(0.118)
***Household characteristics***			
Age of Mother (years)	26.833	29.417	2.583 *
	(3.460)	(3.288)	(1.378)
Maternal education (1 = completed high school or above)	0.250	0.250	0.000
	(0.452)	(0.452)	(0.185)
Mother is primary caregiver (1 = yes)	0.750	0.750	0.000
	(0.452)	(0.452)	(0.185)
Paternal education (1 = completed high school or above)	0.000	0.167	0.167
	(0.000)	(0.389)	(0.112)
Father lived at home during most of last year (1 = yes)	0.750	0.750	0.000
	(0.452)	(0.452)	(0.185)
Asset index (PCA score)	0.007	0.059	0.052
	(2.132)	(1.615)	(0.772)
Number of adults in the household	2.083	2.333	0.250
	(1.084)	(1.073)	(0.440)
Number of siblings at home	0.333	0.167	−0.167
	(0.492)	(0.389)	(0.181)

Standard errors in parentheses; * *p* < 0.1. “CTC” is defined as Conversational Turns Count. “PCA” is defined as Principal Component Analysis. Column (3) represents the value of Column (2) minus Column (1).

**Table 4:** Differences in characteristics between top and bottom terciles of Child Vocalizations Count (N = 38).

Variables	Bottom ⅓ of CVC	Top ⅓ of CVC	Difference
	(1)	(2)	(3) = (2) − (1)
***Child characteristics***			
Age (months)	24.183	24.656	0.473
	(2.168)	(2.041)	(0.842)
Gender (1 = boy)	0.500	0.615	0.115
	(0.522)	(0.506)	(0.206)
Prematurity (1 = yes)	0.083	0.154	0.071
	(0.289)	(0.376)	(0.135)
***Household characteristics***			
Age of Mother (years)	28.667	28.923	0.256
	(4.519)	(3.121)	(1.543)
Maternal education (1 = completed high school or above)	0.167	0.308	0.141
	(0.389)	(0.480)	(0.176)
Mother is primary caregiver (1 = yes)	0.833	0.692	−0.141
	(0.389)	(0.480)	(0.176)
Paternal education (1 = completed high school or above)	0.000	0.231	0.231 *
	(0.000)	(0.439)	(0.127)
Father lived at home during most of last year (1 = yes)	0.833	0.692	−0.141
	(0.389)	(0.480)	(0.176)
Asset index (PCA score)	−0.445	0.407	0.852
	(1.759)	(1.780)	(0.709)
Number of adults in the household	1.833	2.308	0.474
	(1.115)	(1.182)	(0.461)
Number of siblings at home	0.500	0.154	−0.346 *
	(0.522)	(0.376)	(0.181)

Standard errors in parentheses; * *p* < 0.1. “CVC” is defined as Child Vocalization Count. “PCA” is defined as Principal Component Analysis. Column (3) represents the value of Column (2) minus Column (1).

**Table 5:** Multivariate correlations between household characteristics and AWC, CTC and CVC.

Variables	AWC	CTC	CVC
	(1)	(2)	(3)
***Child characteristics***			
Age (months)	−125.62	0.49	82.37
	(649.88)	(26.15)	(79.87)
Gender (1 = boy)	241.00	−5.90	23.24
	(2778.50)	(111.79)	(341.47)
Prematurity (1 = yes)	−2915.19	−36.08	387.04
	(4267.58)	(171.70)	(524.47)
***Household characteristics***			
Age of Mother (years)	288.76	34.10 **	60.32
	(401.61)	(16.16)	(49.36)
Maternal education (1 = completed high school or above)	−1477.58	−94.30	−164.36
	(3487.68)	(140.32)	(428.62)
Mother is primary caregiver (1 = yes)	−4695.00	−157.14	100.65
	(3200.18)	(128.75)	(393.29)
Paternal education (1 = completed high school or above)	4282.59	189.33	253.99
	(5017.59)	(201.87)	(616.64)
Father lived at home during most of last year (1 = yes)	−401.06	92.82	254.68
	(2723.92)	(109.59)	(334.76)
Asset index (PCA score)	98.85	45.04	24.04
	(1079.30)	(43.42)	(132.64)
Number of adults in the household	2,172.71	64.63	93.74
	(1499.87)	(60.34)	(184.33)
Number of siblings at home	−2770.30	−292.05 *	−619.62
	(3572.16)	(143.72)	(439.00)
Observations	38	38	38
R-squared	0.34	0.45	0.32

Source: Authors’ survey. Standard errors in parentheses; ** *p* < 0.05, * *p* < 0.1. “AWC” is defined as Adult Word Count. “CTC” is defined as Conversational Turns Count. “CVC” is defined as Child Vocalization Count. “PCA” is defined as Principal Component Analysis. Column (3) represents the value of Column (2) minus Column (1).

**Table 6:** Multivariate correlations between AWC, CTC, CVC and CDI.

Variables	AWC	CTC	CVC
	(1)	(2)	(3)
***Home Language Environment Measures***			
AWC	0.00		
	(0.00)		
CTC		0.03 **	
		(0.02)	
CVC			0.01 *
			(0.01)
***Child characteristics***			
Age (months)	1.54	1.48	0.72
	(2.08)	(1.87)	(1.99)
Gender (1 = boy)	−4.93	−4.63	−5.06
	(8.87)	(7.99)	(8.30)
Prematurity (1 = yes)	−25.46 *	−25.29 *	−30.20 **
	(13.76)	(12.29)	(12.91)
***Household characteristics***			
Age of Mother (years)	3.22 **	2.14	2.76 **
	(1.30)	(1.27)	(1.24)
Maternal education (1 = completed high school or above)	22.96 *	25.69 **	23.96 **
	(11.18)	(10.14)	(10.46)
Mother is primary caregiver (1 = yes)	−13.53	−9.81	−16.23
	(10.70)	(9.51)	(9.58)
Paternal education (1 = completed high school or above)	5.41	0.42	4.62
	(16.28)	(14.72)	(15.05)
Father lived at home during most of last year (1 = yes)	−1.06	−4.44	−3.61
	(8.70)	(7.96)	(8.24)
Asset index (PCA score)	8.10 **	6.57 *	7.91 **
	(3.45)	(3.18)	(3.23)
Number of adults in the household	−5.45	−6.89	−5.53
	(5.01)	(4.43)	(4.51)
Number of siblings at home	−21.39 *	−12.26	−16.59
	(11.56)	(11.20)	(11.15)
Observations	38	38	38
R-squared	0.63	0.70	0.68

Source: Authors’ survey. Standard errors in parentheses; ** *p* < 0.05, * *p* < 0.1. “AWC” is defined as Adult Word Count. “CTC” is defined as Conversational Turns Count. “CVC” is defined as Child Vocalization Count. “PCA” is defined as Principal Component Analysis.
